# Intelligent Evaluation Method for Scoliosis at Home Using Back Photos Captured by Mobile Phones

**DOI:** 10.3390/bioengineering11111162

**Published:** 2024-11-18

**Authors:** Yongsheng Li, Xiangwei Peng, Qingyou Mao, Mingjia Ma, Jiaqi Huang, Shuo Zhang, Shaojie Dong, Zhihui Zhou, Yue Lan, Yu Pan, Ruimou Xie, Peiwu Qin, Kehong Yuan

**Affiliations:** 1 Institute for Hospital Management of Tsinghua University, Shenzhen 518000, China; lys18@tsinghua.org.cn (Y.L.); sddsj@163.com (S.D.); yuankh@sz.tsinghua.edu.cn (K.Y.); 2 Tsinghua Shenzhen International Graduate School, Shenzhen 518000, China; 3 Huadu District People’s Hospital of Guangzhou, Guangzhou 510899, China; m15202099401@163.com (Q.M.); 15813363368@139.com (Z.Z.); lyueyy@126.com (Y.L.); 4 School of Medicine, Tsinghua University, Beijing 100010, China; zssure@163.com; 5 Beijing Tsinghua Changgung Hospital, Beijing 100010, China; xrma03496@btch.edu.cn

**Keywords:** scoliosis, back image, computer vision, deep learning, home rehabilitation

## Abstract

The traditional scoliosis examination based on X-ray film is not suitable for large-scale screening, and it is also not suitable for dynamic evaluation during rehabilitation. Therefore, based on computer vision technology, this paper puts forward an evaluation method of scoliosis with different photos of the back taken by mobile phones, which involves three aspects: first, based on the key point detection model of YOLOv8, an algorithm for judging the type of spinal coronal curvature is proposed; second, an algorithm for evaluating the coronal plane of the spine based on the key points of the human back is proposed, aiming at quantifying the deviation degree of the spine in the coronal plane; third, the measurement algorithm of trunk rotation (ATR angle) based on multi-scale automatic peak detection (AMPD) is proposed, aiming at quantifying the deviation degree of the spine in sagittal plane. The public dataset and clinical paired data (mobile phone photo and X-ray) are used to test. The results show that this method has high accuracy and effectiveness in distinguishing the type of spinal curvature and evaluating the degree of deviation, which is higher than other deep learning algorithms.

## 1. Introduction

In recent years, scoliosis has become more common in adolescents. If not detected and intervened in a timely manner, it can lead to serious deformities and cause visceral dysfunction [1]. Scoliosis refers to the curvature of the back spine that deviates from the centerline of the trunk on one or both sides, forming an “S” or “C” shape [2]. In the clinic, Cobb angle measurement of X-ray images is the “gold standard” to evaluate the severity of scoliosis in patients. The degree of scoliosis is mainly evaluated by doctors’ manual measurement [3]. However, manual evaluation of the Cobb angle of scoliosis is time-consuming and has errors. The Cobb angle measured manually by the same doctor varies from 3 to 5, while it varies from 5 to 7 among different doctors [4]. For inexperienced evaluators, the error may be higher. Nevertheless, X-ray examination is relatively simple; patients do not need to accept complicated preparation procedures, and the examination process is fast. Compared with other advanced imaging examination methods, X-ray examination has a lower cost and is more suitable as a preliminary screening tool. This makes it still the mainstream screening method at present. But the size of the Cobb angle is directly related to the choice of patients’ braces or the diagnosis and decision of surgery [5]. Therefore, accurate automatic calculation and comprehensive evaluation of the Cobb angle are of great value in clinical practice.

Using computer vision technology and deep learning algorithms can effectively lower the threshold of scoliosis evaluation. At present, the spine X-ray image analysis method based on deep learning has made progress in the automatic evaluation of adolescent idiopathic scoliosis. By training a deep convolution neural network to detect vertebrae or segment vertebrae, the Cobb angle measurement can be realized, which greatly improves the evaluation efficiency of scoliosis severity [6,7]. However, based on X-ray examination, there is still radiation exposure, and it is not flexible in time and space, which limits its role in early screening of adolescent scoliosis and long-term dynamic evaluation of scoliosis patients.

In addition to X-ray films, there are several imaging methods that are considered relatively reliable. Multi-slice spiral Computed Tomography (CT) is a commonly used diagnostic examination method in clinical practice, and its accuracy is higher than that of the X-ray examination. But this method still produces radiation [8]. Magnetic resonance imaging (MRI) technology is non-invasive and has high spatial resolution. It can not only clearly display the anatomical structure of bone, find the abnormal development of the vertebral body and its appendages, but also check whether the spinal cord is abnormal. It is of great value for preoperative diagnosis and postoperative review of scoliosis, but the cost is too high [9]. Three-dimensional (3D) ultrasound technology can conveniently and intuitively display the shape of the spine, realize the visualization of the 3D shape of the spine, and accurately measure the curvature of the coronal plane and sagittal plane, so as to rotate axially to the vertebral body [10].

Many studies have focused on using 3D scanning equipment to evaluate scoliosis. Fiona et al. developed a 3D measurement system, which consists of several high-precision sensors. By using non-contact 3D scanning technology, the 3D shape data of the back surface can be obtained by projecting a grating or a laser beam on the patient’s back and capturing the position information of the reflected light. Based on the extracted feature points, the system can calculate the key parameters, such as the bending angle and rotation degree of the spine, which provides a basis for subsequent evaluation and treatment [11]. Alexander et al. designed a 3D body scanner equipped with an infrared sensor and video camera to capture the patient’s body image. Through biomechanical modeling, the biomechanical models of the rib cage and spinal column are simulated based on the finite element method to calculate and compare the specific spinal deformation of patients. It aims to be used as the supplementary information of Cobb angle for daily clinical evaluation of spinal deformation and may reduce the use of X-rays in a follow-up examination [12].

However, most of the above detection methods need to rely on professional equipment, which does not have the real-time and convenience of screening at home. Therefore, this paper aims to design a deep learning algorithm that can evaluate scoliosis without X-rays, only using the back photos in standing position and bare-back photos taken by mobile phones. The goal of this algorithm is to evaluate the severity of scoliosis more accurately than ordinary human doctors when only the image data of patients’ backs are available. This method can not only save medical resources, improve screening efficiency, and reduce radiation caused by routine X-ray follow-up but also realize home automatic identification of back photos taken by mobile phones and make a long-term, dynamic, and remote evaluation of the patient’s spine, which has important medical, economic, and social values.

The University of Hong Kong developed a virtual spine evaluation platform [13]. The pictures of the human back collected by the mobile phone are used to evaluate the risk of spinal deformity and judge whether there is any progress in spinal deformity. Through three deep learning frameworks based on Visual Transformer, Residual Network, and Attention Mechanism, the platform receives photos as input to analyze the degree of scoliosis. The three-classification model is trained for severity classification, and the two-classification model is trained for the curve-type classification. Xinhua Hospital Affiliated with the Medical College of Shanghai Jiaotong University, proposed a scoliosis hierarchical depth learning algorithm for back photos. The algorithm flow is as follows: First, train the Faster-RCNN network to locate the key areas of the photographed human back photos. Then, for the photos of key back regions, the ResNet50 network is trained to realize four classifications of scoliosis angles (<10, 10–19, 20–44, ≥45). The accuracy of this method in the four classification tasks of scoliosis is 0.555, which is slightly lower than that of ordinary doctors (56.9%) [14]. Subsequently, Xidian University improved the classification method of scoliosis on this basis. Three two-classification models of scoliosis are used to replace the traditional four-classification model, thus realizing a more simplified and effective identification of scoliosis diseases [15].

In structural scoliosis, the spine will be offset not only in the coronal plane but also in the sagittal plane [16]. Therefore, scoliosis must be regarded as a three-dimensional deformity of the spine and trunk, which may deteriorate rapidly during rapid growth. However, at present, the research pays more attention to the interval estimation of the deviation angle of the coronal plane of the spine but ignores the research on the types of spinal curvature and the degree of sagittal plane deviation of the spine. In this paper, a multi-dimensional scoliosis evaluation algorithm is designed and implemented using bare-back photos, including a trunk rotation measurement algorithm, spinal curvature type discrimination algorithm, and spinal coronal scoliosis severity classification algorithm, integrating doctors’ knowledge. Compared with the previous algorithms that only evaluated the degree of coronal deviation of the spine, this set of algorithms has higher reliability and improved the classification accuracy, recall rate, and F1-score. It can also be used for the long-term dynamic evaluation of scoliosis and visually show the changes in scoliosis during rehabilitation treatment.

## 2. Methods

### 2.1. Discrimination Algorithm of Spine Coronal Curvature Type

When the coronal plane of the spine is curved, it will cause the body to appear like “C” or “S” or even be multi-curved in the upright position. In past research work on scoliosis evaluation based on back photos, the differentiation of scoliosis types has not been addressed. But in the clinic, the type of spinal curvature is an important reference index for doctors to evaluate patients’ spinal condition. Therefore, based on the key point detection model of YOLOv8 [17], this paper proposes an algorithm to distinguish the types of coronal curvature of the spine.

#### 2.1.1. Back Key Point Detection

**Figure 1:**
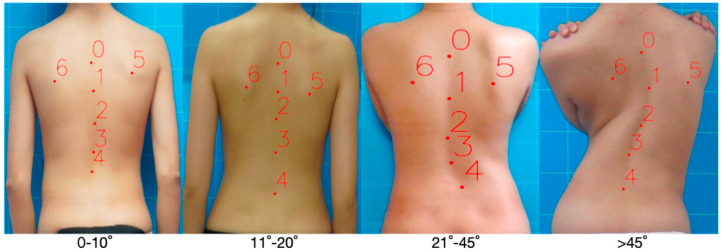
Schematic diagram of marking key points on the back.

Professional doctors were invited to manually mark the key points on the back of 3640 standing half-length photos with LabelMe (version 3.16.7). The key points marked include five key points along the spine, which are marked with serial numbers 0, 1, 2, 3, and 4, respectively, and double scapular points with serial numbers 5 and 6, respectively, as shown in Figure 1.

YOLOv8 has a wide range of visual AI functions, including target detection, attitude estimation, target tracking, and many other tasks [18]. Table 1 presents the loss function of YOLOv8 in the key point detection task.

(1) $$L_{kpts}=1-\sum_{n=1}^{N_{kpts}}\;OKS=1-\frac{\sum_{n=1}^{N_{kpts}}\;exp\;\left(\frac{d_{n}^{2}}{2s^{2}k_{n}^{2}}\right)\delta \left(v_{n}>0\right)}{\sum_{n=1}^{N_{kpts}}\;\delta \left(v_{n}>0\right)}$$

where $d_{n}=$ ${\left({\hat{x}}_{n}-x_{n}\right)}^{2}+{\left({\hat{y}}_{n}-y_{n}\right)}^{2}$ represents the Euclidean distance between the predicted position and the actual position of the nth key point. $k_{n}$ represents the specific weight of the key point. s represents the dimension of key points, and $\delta \left(v_{n}\right)$ represents the visibility sign of each key point.

(2) $$L_{kobj}=-\frac{1}{N}\sum_{i=1}^{N}{𝟙}_{i}^{obj}\left[y_{i}\log\left({\hat{y}}_{i}\right)+\left(1-y_{i}\right)\;\log\left(1-{\hat{y}}_{i}\right)\right]-\;\lambda_{noobj}\frac{1}{N}\sum_{i=1}^{N}\;{𝟙}_{i}^{noobj}\left[y_{i}\log\left({\hat{y}}_{i}\right)+\left(1-y_{i}\right)\log\left(1-{\hat{y}}_{i}\right)\right]$$

In this formula, $y_{i}$ represents the true category of the q-th sample, and ${\hat{y}}_{i}$ represents the prediction probability of the q-th sample. $\lambda_{noobj}$ is the background weight, and users prevent the model from paying too much attention to the background information during training.

(3) $$L_{cls}=-\frac{1}{N}\sum_{i=1}^{N}\;\sum_{n=1}^{N_{kpts}}\;p_{in}log\left({\hat{p}}_{in}\right)$$

$p_{in}$ is an indicator function. When the i sample belongs to the n class, $p_{in}$ is 1. Otherwise, ${\hat{p}}_{in}$ represents the prediction probability of the model that the i sample belongs to the n class.

The total loss function $L_{total}$ is the weighted sum of the three partial loss functions $L_{kpts}$, $L_{kobj}$, and $L_{cls}$, where $\lambda_{kpts}$, $\lambda_{kobj}$, and $\lambda_{cls}$ are the weights of the corresponding loss functions, respectively:

(4) $$L_{total}=\lambda_{kpts}L_{kpts}+\lambda_{kobj}L_{kobj}+\lambda_{cls}L_{cls}$$

#### 2.1.2. Curve Fitting of Key Points of Spine

Curve fitting of key points refers to connecting discrete key points by adopting appropriate mathematical models or functions, thus forming a continuous curve or surface [19]. In this section, the least square method is used to fit the curves of five key points along the detected spine in order to show whether the spine is in an “S” shape or a “C” shape. The specific curve-fitting process of key points along the spine is as follows:

1. Let the fitting polynomial be:

(5) $$\hat{y}=a_{0}+a_{1}x+\cdots +a_{4}x^{4}$$

2. The sum of distances from each point to this curve, that is, the sum of squares of errors:

(6) $$R^{2}=\sum_{i=1}^{5}\;{\left(y_{i}-{\hat{y}}_{i}\right)}^{2}=\sum_{i=1}^{5}\;{\left[y_{i}-\left(a_{0}+a_{1}x+\cdots +a_{4}x^{4}\right)\right]}^{2}$$

3. According to the least square principle, the partial derivative of the right side of the equation is solved as follows:

(7) $$\begin{array}{c} 5a_{0}+a_{1}\sum_{i=1}^{5}\;x_{i}+\cdots +a_{4}\sum_{i=1}^{5}\;x_{i}^{4}=\sum_{i=1}^{5}\;y_{i}\\ a_{0}\sum_{i=1}^{5}\;x_{i}+a_{1}\sum_{i=1}^{5}\;x_{i}^{2}+\cdots +a_{4}\sum_{i=1}^{5}\;x_{i}^{5}=\sum_{i=1}^{5}\;x_{i}y_{i}\\ \cdots \\ a_{0}\sum_{i=1}^{5}\;x_{i}^{4}+a_{1}\sum_{i=1}^{5}\;x_{i}^{5}+\cdots +a_{4}\sum_{i=1}^{5}\;x_{i}^{8}=\sum_{i=1}^{5}\;x_{i}^{4}y_{i} \end{array}$$

4. Into a matrix form

(8) $$\left[\begin{array}{cccc} 1 & x_{1} & \cdots & x_{1}^{4}\\ 1 & x_{2} & \cdots & x_{1}^{4}\\ 1 & x_{3} & \cdots & x_{1}^{4}\\ 1 & x_{4} & \cdots & x_{1}^{4} \end{array}\right]\left[\begin{array}{c} a_{0}\\ a_{1}\\ \vdots \\ a_{4} \end{array}\right]=\left[\begin{array}{c} y_{1}\\ y_{2}\\ \vdots \\ y_{5} \end{array}\right]$$

5. Solve the above equations to obtain the fitting curve expression:

(9) $$\hat{y}=a_{0}+a_{1}x+\cdots +a_{4}x^{4}$$

#### 2.1.3. Identification of Fitting Curve Types for Key Points of Spine

Generally, the “S” curve has two tangent points, while the “C” curve has only one tangent point [20]. Therefore, the type of curve can be determined by analyzing the number of tangent points of the key point fitting curve. The sliding window method is used to find the tangent point on time series or curve data. The specific steps are as follows:

1. Determine the window and step size: assume that the length of the curve sequence is length, define the window size as length/10, and set the step size as 2;
2. Sliding window: from the starting point of the curve, slide the window to the end of the curve. Every time you move a window, you obtain a subset of the data in the window;
3. Calculating the features in the window: for the data subset in each window, the corresponding slope and curvature features are calculated. These characteristics are helpful in describing the curve changes in the window;
4. Judging the tangent point: judging whether the data in the window have the characteristics of showing the tangent point. The characteristic of the tangent point is that the slope changes from positive slope to negative slope or from negative slope to positive slope within the data subset where the point is located;
5. Record the tangent point position: if the tangent point is detected in a certain window, record the tangent point position and the tangent point curvature;
6. Curvature check: the maximum curvature usually appears at the concave point or the convex point of the curve. If the curvature of the tangent point recorded above is greater than that of the other non-tangent points, it will be kept; otherwise, it will be discarded. This scheme is helpful in increasing the credibility of the found tangent point.

Therefore, if there is only one tangent point in the curve found by the sliding window method, it means that the curvature type of the coronal plane of the spine is “C” (single curve). If there are two tangent points, it shows that the curvature type of the coronal plane of the spine is “S” (hyperbolic). If the number of tangent points of the curve found by the sliding window method is more than two, it means that the curvature type of the coronal plane of the spine is multi-curved.

### 2.2. Research on Grading Algorithm of Vertebral Coronal Scoliosis Degree

In the task of grading the degree of scoliosis in the coronal plane based on bare-back photos, the algorithm model receives the posterior photos of the human back as input and outputs four classification results of the Cobb angles of scoliosis (<10, 10–19, 20–44, ≥45). In the past, the Faster-RCNN network was usually used [21]. First, the key areas of the back were located, and then, the classification neural network was trained separately for these key area photos so as to realize the four classifications of the scoliosis angles. However, this scheme has some defects. The classification neural network is usually regarded as a black box model, and its internal decision-making process is difficult to explain.

Therefore, this paper proposes a new method. Based on the doctor’s experience in judging the degree of curvature of the coronal plane of the spine by using the bare-back image of the patient, this method first calculates the Cobb angle of scoliosis based on the curve-fitting of the key points of the spine after detecting the key points of the back photo. Secondly, the absolute value of the bottom corner difference is calculated by using a triangle composed of double scapular points and the endpoints along the spine. With the increase in lateral curvature of the coronal plane of the spine, the difference between the Cobb angle and the bottom corner will also increase accordingly. Using these two characteristic indexes to quantify the degree of scoliosis in the coronal plane of the spine is more interpretable than relying only on the output of the classified neural network.

#### 2.2.1. Calculation of the Cobb Angle of Spine

**Figure 2:**
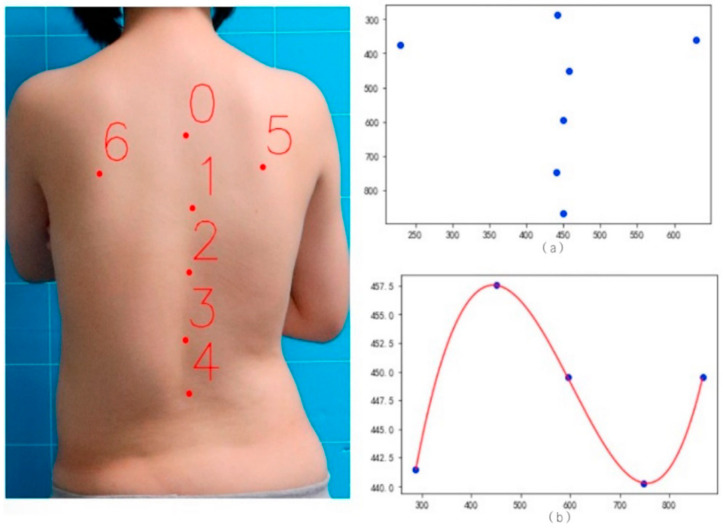
Effect diagram of back key points and spine-fitting curve. (**a**) Key point diagram of spine and scapula. (**b**) Fitting diagram of key points and curves of spine.

Measuring the Cobb angle of the spine [22] is a common method to evaluate scoliosis. Figure 2 shows the key points of the spine and the corresponding fitting curve. The specific steps for calculating the Cobb angle based on the fitting curve of the key points of the spine are as follows:

1. Using the curve type discrimination algorithm proposed in Section 2.1.3, it is determined whether the fitting curve of spine key points belongs to the “C” type (single-curve), the “S” type (hyperbolic curve), or the multi-curve type;
2. For the “C” type (single-curve) scoliosis, the Cobb angle is measured according to the following steps: Divide the curve into two sections: [0, x] and (x, L], where l represents the length of the curve, and x represents the position of the tangent point. In the interval [0, x], find the point with the maximum absolute value of the curve slope, which is determined as the upper vertebra. In the interval (x, L], find the point with the maximum absolute value of the curve slope, which is determined as the lower vertebra. Tangents are made to the upper vertebral point and the lower vertebral point. Calculate the included angle between the two tangents to obtain the Cobb angle;
3. For the S-shaped (hyperbolic) and multi-curved scoliosis, measure the overall curvature of the spine according to the following steps: Analyze the second derivative of the fitting curve to find the position of the inflection point of the curve. Divide the curve into the convex part and the concave part according to the inflection point. Measure the Cobb angle for each part by the method mentioned before. Accumulate the Cobb angle of each part to evaluate the overall curvature of the spine.

#### 2.2.2. Calculation of the Difference Between the Bottom Corners of the “Scapular Spine Triangle”

**Figure 3:**
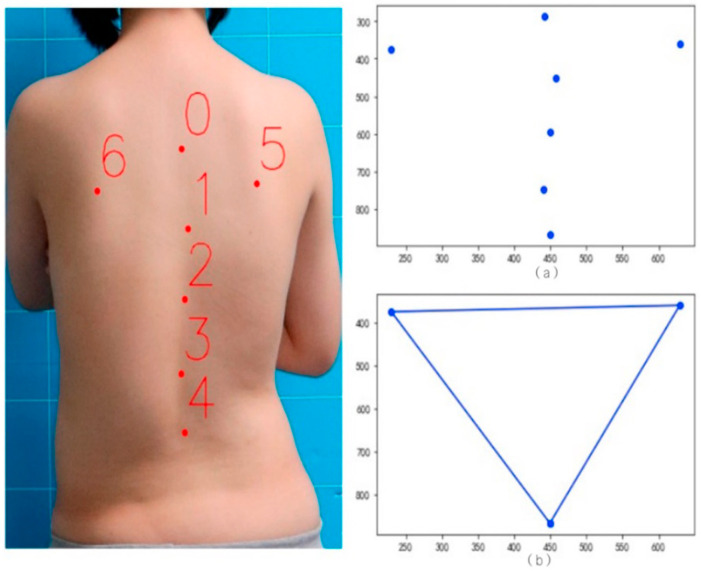
Triangle effect diagram of back key points and scapula spine. (**a**) Key point diagram of spine and scapula. (**b**) Key point diagram of scapula and spinal terminal point.

The triangle formed by the two scapular points and the end of the spine is called the “scapular spine triangle” [23]. This triangle is often used in the fields of medicine and ergonomics, such as the evaluation of body posture and spine. The bottom corner refers to the angle on the bottom edge formed by the point of the double scapula and the end of the spine. Figure 3 shows the back key points and the scapular spine triangle. In this section, the absolute value of the difference between the two corners of the double bottom is calculated to evaluate the bending degree of the spine on the coronal plane.

The calculation steps of the bottom corner difference in the “scapular spine triangle” based on the back key points are as follows:

1. According to the detection results of key points on the back in Section 2.1.1, based on this result, the coordinates of the double scapula points and the spine endpoints are obtained. Then, connect these three points in pairs to form a “scapular spine triangle”;
2. The absolute value of the difference between the two angles can be obtained by calculating the values of the two angles on the bottom of the scapular spine triangle.

### 2.3. Measurement Algorithm of Trunk Rotation in Sagittal Plane of Spine

#### 2.3.1. Algorithm Flow

**Figure 4:**
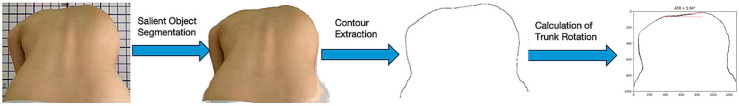
Flow chart of spinal sagittal plane evaluation algorithm.

The flow chart of the measurement algorithm for the sagittal torso rotation of the spine is shown in Figure 4:

1. Image preprocessing stage: preprocess the input image (90-degree bending back photos), including removing noise, adjusting brightness, and enhancing contrast so as to improve the robustness and accuracy of the subsequent algorithm;
2. Salient object segmentation: using salient object segmentation technology, background information is removed from the image, and salient objects in the image are segmented for subsequent processing;
3. Contour extraction: Use the edge detection algorithm to extract the contour from the back image so as to further analyze the shape features of the back;
4. Calculation of trunk rotation: the contour line is analyzed by the automatic multi-scale peak detection algorithm to find the position point of the back bulge, and the deviation degree of the spinal sagittal plane is evaluated by calculating trunk rotation.

#### 2.3.2. Salient Object Segmentation Algorithm

**Figure 5:**
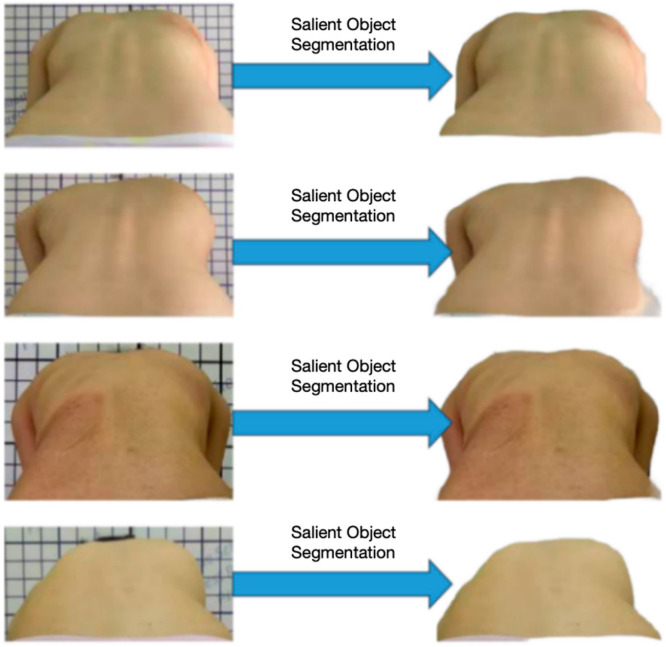
SAM saliency segmentation effect diagram.

In this paper, a new concept of segmentation task, namely, “Hintable segmentation task”, is proposed. The goal of this concept is to make any segmentation hint generate an effective segmentation mask. Therefore, the hintable segmentation task is used as a pre-training target, and the general downstream segmentation task is solved through hint engineering. With the help of the effective generalization ability of the SAM model in subdividing image segmentation tasks [24], this paper can be deployed directly on the server without additional training. By inputting the photographed bitmap of the bent human body into the SAM model, the algorithm can effectively segment the back part of the human body in the image and remove the background information, as shown in Figure 5.

#### 2.3.3. Contour Extraction Algorithm

In this paper, the edge detection algorithm based on the gradient is adopted, and the gradient information in the image is used to find the maximum or the largest change position of the gradient value so as to detect the edge of the target and extract the contour line of the target. Common gradient-based algorithms include Roberts, Prewitt, and LOG operators.

Roberts is an edge detection operator widely used in the field of image processing [25]. Its main function is to find the edge in the image through local difference information. Roberts operator convolves the image with two 2 × 2 convolution kernels, which are used to detect the horizontal edge information $d_{x}(i,j)$ and the vertical edge information $d_{y}(i,j)$ in the image, respectively. The final edge strength of each pixel S(i,j) can be calculated by the following formula:

(10) $$S(i,j)=\sqrt{\left(d_{x}(i,j{)}^{2}+d_{y}(i,j{)}^{2}\right)}$$

Prewitt is a first-order differential operator for image edge detection [26]. This method detects the edge by comparing the gray difference between the pixel and its upper, lower, left, and right neighboring points and produces an extreme value at the edge position. This design is helpful in accurately capturing the edge features in the image while suppressing some false edges. Prewitt operator convolves the image with two 3 × 3 convolution kernels, which are used to detect the horizontal edge information $d_{x}(i,j)$ and vertical edge information $d_{y}(i,j)$ in the image, respectively. Because of the use of convolution kernels in these two directions, the Prewitt operator can smoothen the noise in the image while detecting the edge. The final edge strength of each pixel S(i,j) can be calculated by the following formula:

(11) $$S(i,j)=\sqrt{\left(d_{x}(i,j{)}^{2}+d_{y}(i,j{)}^{2}\right)}$$

LOG is a second-order differential edge detection operator that uses the Laplacian operator to extract image edge information on the basis of the Gaussian function [27]. The approximate formula of its application in digital image processing is

(12) $$\nabla^{2}f(i,j)=f(i+1,j)+f(i-1,j)+f(i,j+1)+f(i,j-1)-4f(i,j)$$

The specific flow of the algorithm is as follows: firstly, the input image is processed by the Gaussian kernel to smoothen the image and remove high-frequency noise. Then, the Laplacian operation is applied to the image to highlight the edges and textures in the image. Finally, the edge in the image is located by detecting the zero crossing point in the result. In order to highlight the back contour information and reduce the interference of other edge information, the image is binarized at first. Then, the Prewitt operator is used for edge detection.

The specific flow of the algorithm is as follows:

- The input image is processed by the Gaussian kernel to smooth the image and remove high-frequency noise;
- Laplacian operation is applied to the image to highlight the edges and textures in the image;
- The edge in the image is located by detecting the zero crossing point in the result.

**Figure 6:**
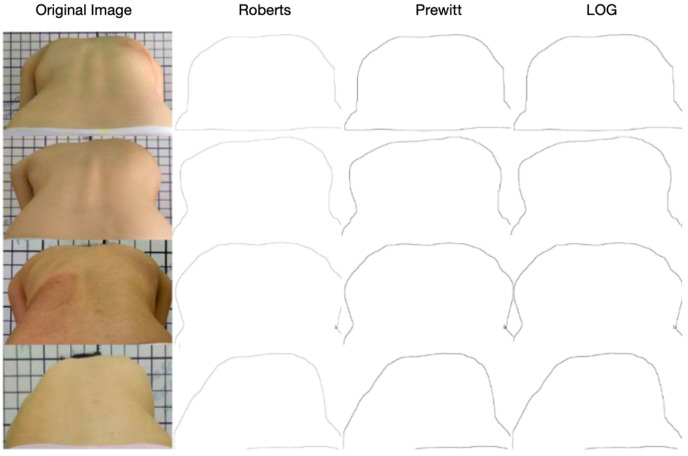
Contour extraction effect diagram of different operators.

In order to highlight the back contour information and reduce the interference of other edge information, the image is binarized at first. Then, Prewitt operator is used for edge detection. The effect of the algorithm is shown in Figure 6:

By comparing the experimental results, it is decided which edge detection operator is the most suitable for extracting the back contour line.

#### 2.3.4. Calculation of Trunk Rotation

In clinical practice, the method of calculating trunk rotation involves finding the double scapular points protruding from the patient’s back and calculating the included angle between the connecting line of the two points and the horizontal line. Therefore, the key difficulty in the calculation of trunk rotation is to accurately find the double scapular points on the contour line of the back, that is, the detection of double peaks.

Felix proposed an automatic multi-scale peak detection algorithm (AMPD) [28]. The algorithm uses multi-scale technology to detect all local maxima of signals and automatically analyzes the results of multi-scale technology to find the “real peak”, thus improving the versatility of the algorithm. The characteristics of the AMPD algorithm are as follows: users d not need to adjust hyperparameters. The peak value in periodic and quasi-periodic signals can be detected. It is relatively robust to high and low-frequency noise.

The principle of the algorithm is as follows: Let $x=\left[x_{1},x_{2},\ldots ,x_{i},\ldots ,x_{N}\right]$ be a given univariate uniform sampling signal, which contains periodic or quasi-periodic peak components.

- The first step of the automatic peak detection (AMPD) algorithm based on multi-scale includes calculating the local maximum scale map (LMS). Therefore, firstly, the signal x is linearly detrended; that is, the least square fitting line of the signal **x** is calculated. Then, the sliding window method is used to determine the local maximum value of signal x. Where the window length $w_{k}$ obeys $\left\{w_{k}=2k|k=1,2,\ldots ,L\right\}$, where k represents the kth scale of the signal; L=⌈N/2⌉−1, and the symbolic function ⌈z⌉ refers to the smallest integer not less than z. For each scale k and i=k+2,…,N−k+1, according to the formula:

(13) $$m_{k,i}=\left\{\begin{array}{cc} 0, & x_{i-1}>x_{i-k-1}\wedge x_{i-1}>x_{i+k-1}\\ r+\alpha , & \mathrm{otherwise} \end{array}\right.$$

In this formula, r is a random number that follows a uniform distribution in the range [0, 1], and α is a constant factor (α=1). For i=1,…,k+1, and for i=N−k+2,…,N, assign the result of r+α to $m_{k,i}$. Expand the above formula in matrix form as follows:

(14) $$M=\left(\begin{array}{cccc} m_{1,1} & m_{1,2} & \ldots & m_{1,N}\\ m_{2,1} & m_{2,2} & \ldots & m_{2,N}\\ \vdots & \vdots & \ddots & \vdots \\ m_{L,1} & m_{L,2} & \ldots & m_{L,N} \end{array}\right)=\left(m_{k,i}\right)$$

where the k-th row contains the value w of the window length $w_{k}$, so all elements of the L×N matrix M are in the range of [0,1+α]. The matrix M is called the LMS of the signal **x**;

- The second step of the algorithm is to sum the LMS matrix M line by line:

(15) $$\gamma_{k}=\sum_{i=1}^{N}\;m_{k,i},\;for\;k\in \{1,2,\ldots ,L\}$$

- Vector $\gamma =\left[\gamma_{1},\gamma_{2},\ldots ,\gamma_{i},\ldots ,\gamma_{L}\right]$ contains the scale-dependent distribution information of local maxima. Global maximum $\lambda ,\lambda =argmin\left(\gamma_{k}\right)$, indicating the scale with the largest local maximum. The value λ is used in the third step of the algorithm, and the LMS matrix M is reshaped by deleting all the elements that k>λ holds, thus generating a new λ×N matrix $M_{r}=\left(m_{k,i}\right)$, for i∈{1,2,…,N} and k∈{1,2,…,λ};
- In the last step of the algorithm, the peak value is detected by calculating the column-by-column standard deviation of the matrix $M_{r}$, and according to the formula:

(16) $$\sigma_{i}=\frac{1}{\lambda -1}\sum_{k=1}^{\lambda }\;{\left[{\left(m_{k,i}-\frac{1}{\lambda }\sum_{k=1}^{\lambda }\;m_{k,i}\right)}^{2}\right]}^{\frac{1}{2}},for\;i\in \{1,2,\ldots ,N\}$$

**Figure 7:**
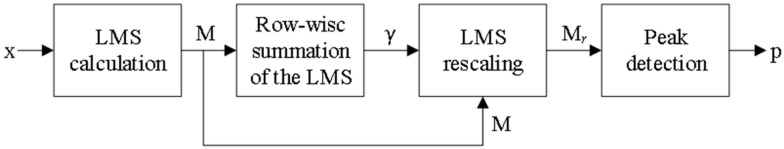
Calculation step diagram of AMPD algorithm.

Find the i that makes $\sigma_{i}=0$ hold in the above formula, and store these values in vector $p=\left[p_{1},p_{2},\ldots ,p_{q},\ldots p_{\hat{N}}\right]$, where $\hat{N}$ represents the total number of peaks detected by signal x, and p represents the index of detected signal peaks. According to the experience, for quasi-periodic input signals, the highest frequency $\left(f_{max}\right)$ should not be more than four times the lowest frequency $\left(f_{min}\right)$, that is, $f_{max}<4f_{min}$. The framework of the AMPD algorithm is shown in Figure 7. For each given signal $x=\left[x_{1},x_{2},\ldots ,x_{i},\ldots ,x_{N}\right]$, the peak is detected by calculating the column-by-column standard deviation of matrix $M_{r}$, so that i where $\sigma_{i}=0$ holds is the peak index.

**Figure 8:**
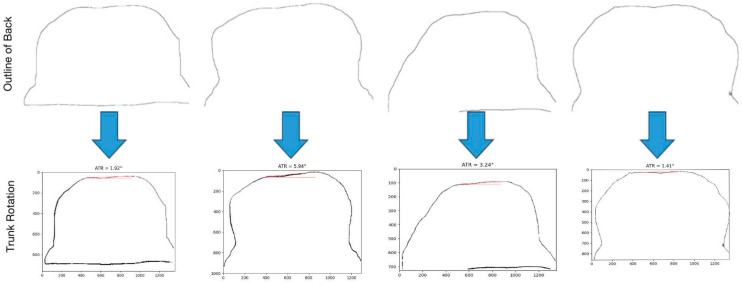
Effect diagram of torso rotation calculation.

According to the AMPD algorithm, this section realizes the peak detection of the back contour. If the algorithm detects more than two peaks, the detection result is processed by filtering. Considering the distance between adjacent peak points and the “flatness” of the area around the peak point, only two peaks are retained. Connect the bimodal points and calculate the angle between them and the horizontal line, which is the rotation degree of the trunk. The effect of the algorithm is shown in Figure 8:

## 3. Results and Discussion

### 3.1. Effect Evaluation of the Algorithm for Judging the Type of Coronal Curvature of the Spine

#### 3.1.1. Training Results of Back Key Point Detection Model Based on YOLOv8

**Figure 9:**
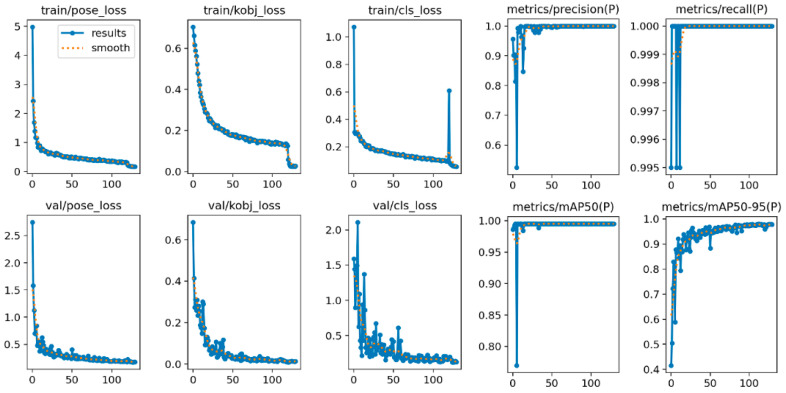
Curve of loss function and evaluation index during model training.

Divide the dataset of 3640 pictures marked by doctors. The data set division method is shown in Table 2:

The evaluation indexes used by the model in the verification set and test set include Precision, Recall, F1 score, map @ 0.5 (mean average precision at iou 0.5), and map @ 0.5: 0.95 (mean average precision from iou 0.5 to 0.95). The complete training results are shown in Figure 9. It is observed that after 130 rounds of training, the loss function gradually tends to be stable. The accuracy rate, recall rate, and mAP improved steadily and finally approached 1, showing the stability of the model performance in the process of continuous optimization.

**Figure 10:**
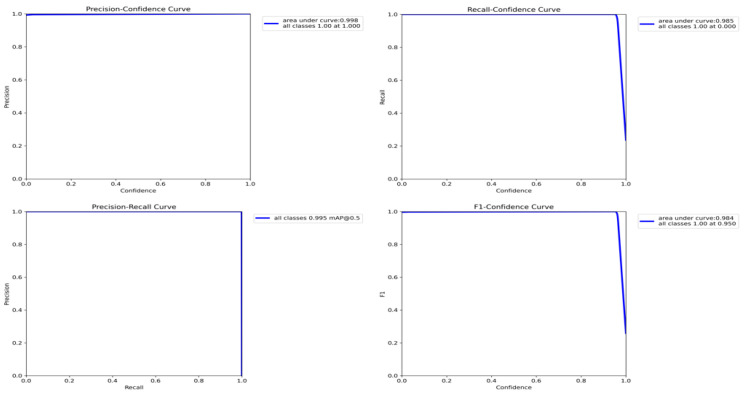
Curve of model test evaluation index.

Performance evaluation of the back key point detection model based on YOLOv8: through the evaluation of the test set, the generalization ability of the model on untouched data is demonstrated. The evaluation indexes include precision rate, recall rate, F1 score curve, and PR curve under different confidence thresholds and mAP@0.5. The evaluation results are shown in Figure 10:

When the confidence threshold is 1.00, the maximum accuracy rate is 1.00. When the confidence threshold is 0.000, the maximum recall rate is 1.00. When the confidence threshold is 0.95, the F1 score reaches the maximum value of 1.00. The relationship between the evaluation index and the area under the curve is shown in Table 3.

It can be observed that the model performs well in the back key point detection task, showing high accuracy. Its mAP score also reaches 0.995, which shows the reliability and generalization ability in practical application.

#### 3.1.2. Effect Evaluation of Bending Type Discrimination Algorithm

Under the guidance of professional doctors in Tsinghua Chang Gung Hospital, we marked the types of spinal curvature on these 3640 back photos, including “C” type (single), “S” type (hyperbolic), and multi-curve type. The specific dataset information is shown in Table 4:

Introduction of evaluation indexes: In order to evaluate the effect of the algorithm for judging the type of spinal coronal curvature, the evaluation indexes selected in this section are classification Accuracy, Precision, Recall, F1 Score, and Kappa Coefficient [29].

1. The calculation formula for Accuracy is as follows:

(17) $$\begin{array}{rr} Accuracy & =\frac{TP+TN}{TP+TN+FP+FN} \end{array}$$

It is mainly calculated by four basic indicators: TP (True Positive); FP (False Negative); FN (False Negative); and TN (true negative);

2. The calculation formula for Precision is as follows:

(18) $$\begin{array}{rr} Precision & =\frac{TP}{TP+FP} \end{array}$$

3. The calculation formula for Recall is as follows:

(19) $$\begin{array}{rr} Recall & =\frac{TP}{TP+FN} \end{array}$$

4. The calculation formula for F1 Score is as follows:

(20) $$F1=\frac{2*Precision*Recall}{Precision+Recall}$$

5. The calculation formula for Kappa Coefficient [21] is as follows:

(21) $$K=\frac{p_{o}-p_{e}}{1-p_{e}}$$

In the formula, $p_{o}$ refers to the actually observed classification accuracy, and $p_{e}$ refers to the classification accuracy when the classifier is randomly predicted. The value range of Kappa Coefficient is between −1 and 1, and the closer to 1, the closer the classifier is to the actual label. Analysis of evaluation results is shown in Table 5.

According to the analysis results, the algorithm proposed in this paper is outstanding in the task of identifying the types of spinal coronal curvature, and the classification accuracy rate reaches 88.7%.

### 3.2. Evaluation of the Effect of Grading Algorithm for Scoliosis in the Coronal Plane of the Spine

Dataset introduction: 3640 half-length photos of standing on the back of the team of Xinhua Hospital Affiliated with the Medical College of Shanghai Jiaotong University, and each photo is marked with its corresponding Cobb angle range by a professional doctor.

#### 3.2.1. Results of Algorithm Based on Curve Fitting of Spine Key Points

In order to further explore the effect of the Cobb angle measurement algorithm based on the fitting curve of spine key points proposed in this paper in the task of grading the degree of scoliosis in the coronal plane, we used this algorithm to grade the degree of scoliosis in 3640 back photos. Analysis of evaluation results is shown in Table 6, Table 7, Table 8 and Table 9.

According to the analysis results, the Cobb angle measurement algorithm proposed in this paper based on the fitting curve of spine key points is outstanding in the task of grading the degree of scoliosis in the coronal plane of the spine. Under the same dataset conditions, compared with the deep learning algorithm of scoliosis classification proposed by the team of Xinhua Hospital Affiliated with the Medical College of Shanghai Jiaotong University, the classification accuracy of the algorithm proposed in this paper is improved by 17.0%. Compared with the screening algorithm of scoliosis based on the convolutional neural network proposed by the team of Xidian University, the classification accuracy is improved by 7.6%. In addition, compared with the above-mentioned method of directly training classified neural networks to identify back photos, the Cobb angle measurement algorithm proposed in this paper is more interpretable because it integrates the clinical experience of doctors.

#### 3.2.2. The Results of the Algorithm Based on the Difference Between the Two Corners of the “Scapular Spine Triangle”

In order to further explore the effect of the absolute value method based on the difference between the two corners of the “scapular spine triangle” proposed in this paper in the task of grading the degree of coronal scoliosis, we used this algorithm to grade the degree of coronal scoliosis of 3640 back photos and also used the classification accuracy, precision, recall, F1 Score, and Kappa Coefficient as evaluation indicators. Analysis of evaluation results is shown in Table 10, Table 11, Table 12 and Table 13:

According to the analysis results, the absolute value method based on the difference between the two bottom corners of the “scapular spine triangle” proposed in this paper performs well in the task of grading the degree of scoliosis in the coronal plane of the spine. Under the same dataset conditions, compared with the deep learning algorithm of scoliosis classification proposed by the team of Xinhua Hospital Affiliated with the Medical College of Shanghai Jiaotong University, the classification accuracy of the algorithm proposed in this paper is improved by 18.4%. Compared with the screening algorithm of scoliosis based on the convolutional neural network proposed by the team of Xidian University, the classification accuracy is improved by 9.0%. In addition, this algorithm is more reliable because it combines the clinical knowledge of doctors. However, the effect of the algorithm is unstable in different types of scoliosis. It performed well in the “C” scoliosis grading task, but it performed poorly in the “S” and multi-curvature scoliosis grading tasks.

#### 3.2.3. Fusion Method

**Figure 11:**
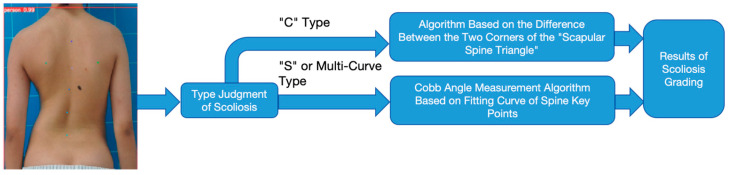
Flow chart of graded fusion method for coronal scoliosis of spine.

According to the analysis of the evaluation results of the above two methods, we found that when dealing with the “C” scoliosis task, the absolute value method of the difference between the two bottom corners of the “scapular spine triangle” was better than the Cobb angle measurement algorithm based on the fitting curve of the spine key points, and the accuracy was improved by 10.9%. However, on the contrary, for the “S” and multi-curve scoliosis tasks, the Cobb angle measurement algorithm based on the curve fitting of spine key points has higher accuracy compared with the absolute value method of the difference between the two bottom corners of the “scapular spine triangle”. Therefore, in order to give full play to the advantages of these two methods, this paper proposes a fusion method on this basis, and the specific content is shown in Figure 11.

The process of the fusion method is as follows: based on the detection results of YOLOv8 back key points, firstly, the scoliosis type is determined by using the spinal curvature type discrimination algorithm. If the discrimination result is a “C”-type scoliosis, the degree of scoliosis is graded by using the absolute value method based on the difference between the two corners of the “scapular spine triangle”; if the discrimination result is “S”-type or multi-curve scoliosis, the Cobb angle measurement algorithm based on the fitting curve of the spinal key points is used. Table 14 shows the overall accuracy of the fusion method and other methods for scoliosis grading tasks based on back photos.

It can be observed that the accuracy of the fusion method is obviously higher than other methods. Moreover, the fusion method makes full use of doctors’ diagnostic knowledge of scoliosis, which makes the algorithm easier to explain. This feature makes this algorithm have high feasibility and practicability in the practical application of home evaluation of spinal coronal scoliosis, and can be widely used in practical scenes.

#### 3.2.4. Verification of Clinical Matched Data

**Figure 12:**
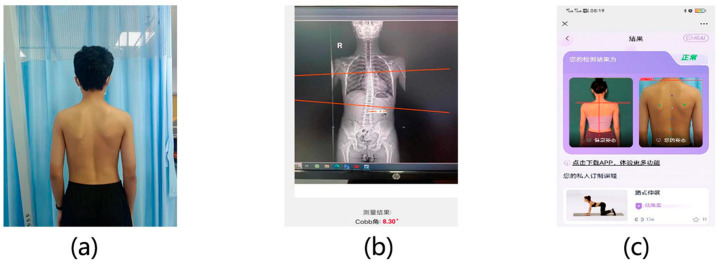
Clinical matched data and analysis results. (**a**) A photograph of the patient’s back. (**b**) The corresponding X-ray of the patient’s back. (**c**) Analysis results of mobile phones integrated into APP.

This study collected the back photos in standing position and X-rays of 25 patients in the hospital. Each patient is marked by a professional doctor with its corresponding Cobb angle range. After comparison, the accuracy of the algorithm proposed in this study reaches 0.702. The specific content is shown in Figure 12:

### 3.3. Effect Evaluation of Spine Spine Sagittal Trunk Rotation Measurement Algorithm

The dataset of 90-degree backward bending photos used in this paper comes from many sources, including online collection, Guangdong Xinmiao Scoliosis Prevention Center, and Beijing Tsinghua Chang Gung Hospital. Under the guidance of doctors, we selected 50 high-quality 90-degree bent back photos to evaluate the effect of the measurement algorithm of spinal sagittal torso rotation. Doctors calculated the torso rotation (ATR angle) of these 50 photos according to clinical standards.

In order to explore the effect of Roberts, Prewitt, and LOG operators in extracting the contour line of the back, this paper uses these three operators to calculate the trunk rotation of 50 photos in the proposed algorithm for measuring the trunk rotation in the sagittal plane of the spine. In order to measure the error between the algorithm measurement results and the doctor’s annotation results, we choose the mean relative error (MRE) as the evaluation index, and its calculation formula is as follows:

(22) $$MRE=\frac{1}{N}\sum_{i=1}^{N}\;\left|\frac{{atr}_{i}-{a\hat{t}r}_{i}}{{atr}_{i}}\right|*100\%$$

${atr}_{i}$ is the value marked by the doctor; ${a\hat{t}r}_{i}$ is the measured value by the algorithm, and *N* is the number of samples. The calculation results are shown in Table 15:

The experimental results show that the Prewitt operator is the most suitable for extracting the contour line of the back in the measurement algorithm of trunk rotation in the sagittal plane of the spine. This choice can minimize the average relative error to only 6.2%. This result shows that the algorithm has high accuracy in evaluating the sagittal curvature of the spine.

## 4. Conclusions

For adolescent idiopathic scoliosis, we should find it as soon as possible and cooperate with doctors for conservative intervention when the Cobb angle is the smallest and flexibility is the best. In this paper, a new method, which does not need X-rays, is proposed, and a deep learning algorithm can be used to evaluate scoliosis in multiple dimensions only by taking bare photos of patients’ backs with mobile phones, which can accurately evaluate the severity of scoliosis, even better than the judgment of ordinary doctors. This method can realize automatic assessment of scoliosis at home, which can not only save medical resources but also reduce the frequency of patients receiving X-ray radiation.

The deep learning algorithm for multi-dimensional evaluation of scoliosis using bare-back photos proposed in this paper is different from previous studies that only focus on the coronal offset angle of the spine. In this paper, the trunk rotation measurement algorithm and the spine bending type discrimination algorithm are proposed for the first time. At the same time, combined with the doctor’s evaluation experience of scoliosis, this paper also puts forward an algorithm for grading the degree of scoliosis in the coronal plane. The classification accuracy of this algorithm exceeds the existing methods and is more interpretable.

In the future, the multi-dimensional scoliosis assessment algorithm can be deployed and applied, and an application program can be developed so that patients can upload pictures with one click and obtain scoliosis assessment results. With the continuous development of telemedicine technology, the following research will focus on establishing a remote monitoring and management system based on intelligent technology. Patients can carry out rehabilitation training through smart devices such as mobile phones and communicate with doctors in real time through the remote monitoring system. Doctors can keep abreast of patients’ rehabilitation progress and provide guidance, thus achieving more flexible and convenient rehabilitation management.

## Figures and Tables

**Table 1:** The loss function of YOLOv8 in the key point detection task.

Loss Function	Description
$L_{kpts}$	When the key point is predicted, the loss function describes the coordinate offset of the key point.
$L_{kobj}$	When forecasting the foreground and background, calculate the cross entropy loss function of the confidence of key points.
$L_{cls}$	In the prediction of key points, cross-entropy loss function is usually used to calculate the loss function of category probability.

**Table 2:** Dataset partition.

Dataset	Total Dataset	Training Set	Verification Set	Test Set
Size/Sheet	3640	2184	728	728

**Table 3:** Evaluation index.

Index	Precision	Recall	F1 Score	PR Curve
Area under curve	0.998	0.985	0.950	0.995

**Table 4:** Information table of spine bending type dataset.

Cobb Angular Range	“C”/Sheet	“S”/Sheet	Multi-Curve/Sheet	Volume
0–10°	503	301	41	845
11–20°	612	327	99	1038
21–45°	472	302	106	880
>45°	313	350	214	877
Volume	1900	1280	460	3640

**Table 5:** Overall evaluation results of discriminant algorithm for spinal coronal bending type.

Bending Type	Precision	Recall	F1 Score	Accuracy	Kappa
“C”	0.942	0.903	0.922	0.887	0.809
“S”	0.843	0.880	0.861		
Multi-curve	0.798	0.839	0.818		

**Table 6:** Evaluation results of “C” scoliosis classification algorithm based on Cobb angle.

Range	Precision	Recall	F1 Score	Accuracy	Kappa
0–10°	0.775	0.726	0.750	0.716	0.615
11–20°	0.684	0.706	0.695		
21–45°	0.699	0.680	0.689		
>45°	0.716	0.773	0.743		

**Table 7:** Evaluation results of “S” scoliosis classification algorithm based on Cobb angle.

Range	Precision	Recall	F1 Score	Accuracy	Kappa
0–10°	0.807	0.777	0.792	0.748	0.621
11–20°	0.750	0.743	0.746		
21–45°	0.688	0.758	0.721		
>45°	0.754	0.717	0.735		

**Table 8:** Evaluation results of multi-curve scoliosis classification algorithm based on Cobb angle.

Range	Precision	Recall	F1 Score	Accuracy	Kappa
0–10°	0.532	0.610	0.568	0.700	0.569
11–20°	0.660	0.707	0.683		
21–45°	0.581	0.708	0.638		
>45°	0.854	0.710	0.775		

**Table 9:** The overall evaluation results of the classification algorithm based on Cobb angle.

Range	Precision	Recall	F1 Score	Accuracy	Kappa
0–10°	0.772	0.738	0.755	0.725	0.632
11–20°	0.702	0.718	0.710		
21–45°	0.679	0.710	0.694		
>45°	0.760	0.735	0.747		

**Table 10:** Evaluation results of classification algorithm for “C” scoliosis based on scapular spine triangle.

Range	Precision	Recall	F1 Score	Accuracy	Kappa
0–10°	0.910	0.805	0.854	0.825	0.763
11–20°	0.797	0.873	0.833		
21–45°	0.809	0.754	0.781		
>45°	0.791	0.872	0.830		

**Table 11:** Evaluation results of classification algorithm for “S” scoliosis based on scapular spine triangle.

Range	Precision	Recall	F1 Score	Accuracy	Kappa
0–10°	0.733	0.674	0.702	0.693	0.590
11–20°	0.661	0.657	0.659		
21–45°	0.600	0.689	0.641		
>45°	0.670	0.631	0.650		

**Table 12:** Evaluation results of grading algorithm for multi-curvature scoliosis based on scapular spine triangle.

Range	Precision	Recall	F1 Score	Accuracy	Kappa
0–10°	0.446	0.610	0.515	0.596	0.433
11–20°	0.536	0.596	0.564		
21–45°	0.489	0.651	0.558		
>45°	0.791	0.565	0.659		

**Table 13:** The overall evaluation results of classification algorithm based on scapular spine triangle.

Range	Precision	Recall	F1 Score	Accuracy	Kappa
0–10°	0.814	0.749	0.780	0.739	0.651
11–20°	0.731	0.778	0.754		
21–45°	0.681	0.719	0.699		
>45°	0.743	0.701	0.721		

**Table 14:** Comparison of classification accuracy.

Classification Method	Triangle of Scapular Spine	Cobb Angle Measurement	Fusion Method	Xinhua Hospital	Xidian University	General Doctor
Accuracy	0.739	0.725	0.793	0.555	0.649	0.569

**Table 15:** The Evaluation Results of Spine Sagittal Trunk Rotation Measurement Algorithm.

Edge Detection Operator	Average Relative Error
Roberts	10.3%
Prewitt	6.2%
LOG	8.4%

## Data Availability

Dataset available on request from the authors.
